# Parallel fabrication of spiral surface structures by interference pattern of circularly polarized beams

**DOI:** 10.1038/s41598-018-31834-3

**Published:** 2018-09-07

**Authors:** Yoshiki Nakata, Masataka Yoshida, Noriaki Miyanaga

**Affiliations:** Institute of Laser Engineering, Osaka University, 2-6 Yamadaoka, Suita, 565-0871 Japan

## Abstract

Mass migration of photo-isomeric azo-polymers occurs according to the light intensity gradient, and the morphological surface structure can be fabricated by the artificial distribution of light by applying the interference properties of coherent laser light. Recently, the optical radiation force has played an important role in the morphology for dielectric targets, and chiral structures have been fabricated according to the spirally gathering force distribution that arises due to the electric susceptibility. On the contrary, interference laser processing has been applied to process the surface or interior of the material, and nano- or micro-structures in the lattice have been fabricated in a single exposure to the interference pattern. The unit structures are mostly axisymmetric nanowhiskers, nanodrops and nanobumps, among others. In this experiment, interference laser processing of an azo-polymer dielectric target using a circularly polarised continuous-wave (CW) laser was examined, and a spiral structure was successfully fabricated. From the viewpoint of laser processing method, an optical spiral radiation force was introduced in interference laser processing for the first time.

## Introduction

The mass migration of photo-isomeric azo-polymers occurs according to the light gradient, and surface-relief gratings have been inscribed by the interference pattern of a linearly polarised beam^1–3^. The transformation mechanism is primarily associated with the periodic shrinkage and back according to *trans–cis–trans* isomerisation cycles by an interference pattern. On the contrary, several studies have reported the fabrication of chiral structures of polymers, metals and semiconductors using an optical vortex with a helical wavefront^4–8^. A detailed experiment using the parameters of momentum *J* = *l* + *s*, where the total angular momentum “*J*” is defined as the sum of the orbital momenta “*l*” and angular spin “*s*”, has been performed for a metal target^9^.

In this case, the spiral frequency was primarily caused by *l* and to a lesser extent by *s*, and no spiral structure could be seen with *l* = 0^7^. However, spiralling on an azo-polymer using a circularly polarised Gaussian beam $(l=0,|s|=1)$ has been successfully demonstrated^9,10^,where the optical radiation force distribution on a dielectric target irradiated using a Gaussian beam was mathematically derived, and a spirally gathering force distribution similar to a cyclone was visually observed.

In past interference femtosecond laser processing experiments, metal lattice nanowhiskers^11,12^ and nanodrops^13,14^, grating^15,16^, embedded grating inside an active medium^17^ and a photonic crystal device^18^ have been fabricated; however, any spiral formations on these nanostructures have not been observed yet. If spirality and chirality could be accomplished using this method, a number of spiral and chiral structures could be fabricated in precise lattice simultaneously, and the effect of the spirality or chirality would be greatly enhanced, leading to an acceleration in the investigation and practical application of these structures.

With respect to applications, chiral or spiral structures have been applied to asymmetric transmission filters^19,20^ and polarisation control^21^ and would be useful for colorimetric chiral recognition of molecule chirality^22^. Most investigations are still in the fundamental research and discovery phases. Moreover, the fabrication techniques being used are lithography and figuring using focused-ion-beam, which are time-consuming and costly; therefore, a new fabrication scheme is needed.

This study aims to demonstrate spiral structure fabrication on azo-polymer film using interference pattern processing with a circularly polarised beam. In the simulation, the similarity of the power distribution between a 6-beam interference pattern and a Gaussian beam is shown, and these optical radiation force distributions which form the spiral structure are discussed. The simulation is also performed for a linearly polarised beam. From the viewpoint of laser processing, optical spiral radiation force was introduced in interference laser processing for the first time.

## Theory

**Figure 1:**
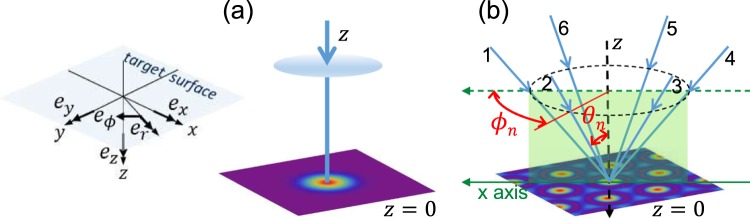
Schemes of the irradiation of (**a**) Gaussian beam and (**b**) interference pattern. The left sketch explains the Cartesian and cylindrical coordinate system and unit vectors.

Figure 1 presents a schematic of the irradiation of (a) a Gaussian beam and (b) an interference pattern. In the latter condition, the axes of the beams form a six-sided pyramid. The left schematic is the coordinate system, where *e*_*x*_, *e*_*y*_ and *e*_*z*_ are the unit vectors of the Cartesian coordinate system, and *e*_*r*_ and *e*_*ϕ*_ are the vectors of the cylindrical coordinate system. For the Gaussian beam, the optical radiation force distribution, which induces the surface-relief structure, is derived mathematically for circularly and linearly polarised beams^9^. On the contrary, the formula for the interference pattern has not yet been derived. Here, the cross-sectional power distribution of a Gaussian spot and a spot in an interference pattern are compared by numerical simulation.

**Figure 2:**
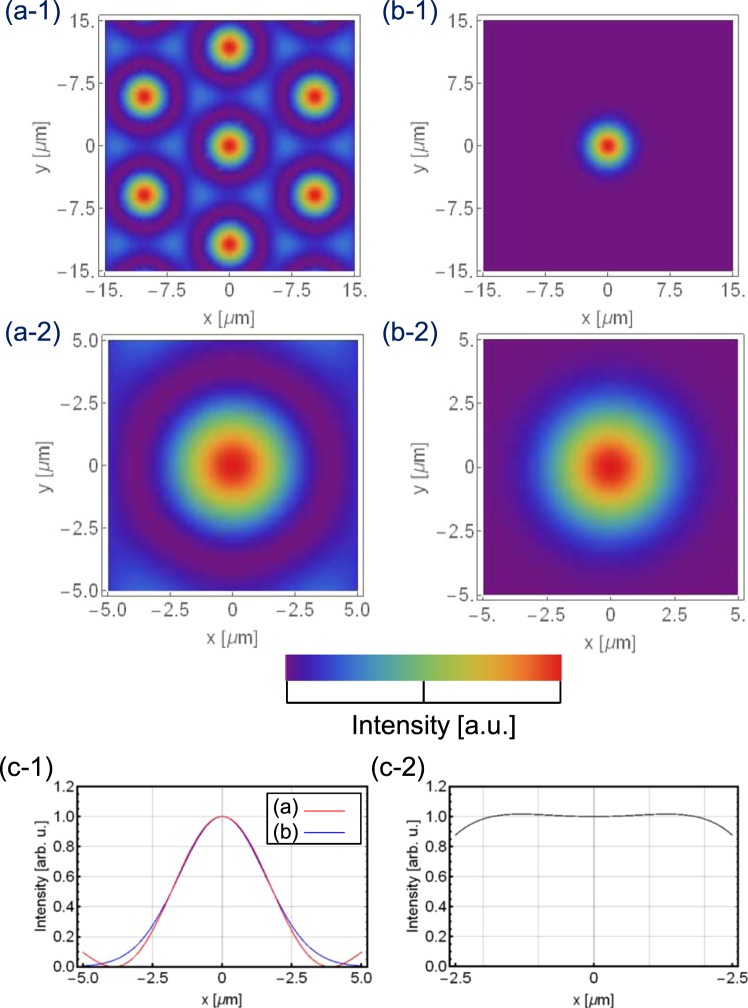
(**a-1**) (**a-2**) Six-beam interference pattern, with wavelength *λ* = 488.0 nm and correlation angle *θ* = 0.0477 rad; (**b-1**) (**b-2**) Gaussian beam, with spot size *ω*_0_ = 2.20 μm; (**c-1**) cross-sectional graph of (**a-2**) and (**b-2**); and (**c-2**) intensity ratio between (**a-2**) and (**b-2**).

To presume the optical radiation force distribution in the interference pattern shown in Fig. 2, the interference pattern used in this experiment was calculated as follows. The electric fields of each beam shown in Fig. 1(b) can be expressed as follows:

1 $$\begin{array}{c}{E}_{n}({E}_{n0},x,y,z,{k}_{n},{\theta }_{n},{\varphi }_{n},{\alpha }_{n},{\omega }_{n},t)\\ \,=\,{E}_{n0}\,\cos \{{k}_{n}(-x\,\sin \,{\theta }_{n}\,\cos \,{\varphi }_{n}-y\,\sin \,{\theta }_{n}\,\sin \,{\varphi }_{n}+z\,\cos \,{\theta }_{n})-{\omega }_{n}t+{\alpha }_{n}\},\end{array}$$

where *E*_*n*0_ represents the electric field of the beams, *k*_*n*_ is the wavenumber, *θ* = *θ*_*n*_ is the polar angle (correlation angle), *ϕ*_*n*_ is the azimuthal angle in the x–y plane, *α*_*n*_ is the phase shift between the beams and *ω*_*n*_ = 2*πc*/*λ*_*n*_ is the corresponding angular frequency. The suffixes 1–6 denote the beam number, as shown in Fig. 1(b). The axes form a six-sided pyramid, so *ϕ*_*n*_ − *ϕ*_*n*−1_ = *2π*/6. Next, the power distribution of an interference pattern of coherent plane wave beams is expressed on the basis of the superposition principle of electric fields^23,24^:

2 $$I({E}_{n0},x,y,z,{k}_{n},{\theta }_{n},{\varphi }_{n},{\alpha }_{n},{\omega }_{n})\propto {\int }^{}{|\sum _{n=1,2,\cdots }^{N}{E}_{n}({E}_{n0},x,y,z,{k}_{n},{\theta }_{n},{\varphi }_{n},{\alpha }_{n},{\omega }_{n},t)|}^{2}dt$$

To simulate an interference pattern, Equation (2) is integrated over Δ*t* = *λ*/*c*. In this simulation, *α*_*n*_ = 0 and *E*_*n*0_ = *E*_0_ are held constant for all the six beams according to the experimental condition.

Figure 2(a-1) and (a-2) depict the interference pattern of the six beams on a target surface (z = 0) with different magnifications. According to the experimental results, the wavelength is *λ* = 488.0 nm, correlation angle is *θ*_int_ = 0.04777 rad and period of the lattice is *Λ* = 11.8 μm.

In the case of the Gaussian beam, the field intensity can be expressed as follows:

3 $${E}_{{\rm{G}}}(r)\propto \exp (-\frac{{r}^{2}}{{\omega }_{0}^{2}})=\exp (-\frac{{x}^{2}+{y}^{2}}{{\omega }_{0}^{2}}).$$

Figure 2(b-1) and (b-2) explain the Gaussian beam, where the spot size is *ω*_0_ = 2.20 μm. Figure 2(c-1) explains the cross-sectional graph of (a-2) and (b-2). The ratio of the intensities is plotted in Fig. 2(c-2), and the difference in spot size is under ±3%.

In the following simulation, the optical radiation force distribution of the Gaussian profile is assumed for each spot in the interference pattern. The optical radiation force of a circularly polarised Gaussian beam is expressed by the following formula for the cylindrical coordinate system^9^:

4 $${\boldsymbol{F}}(r,\varphi )\propto \frac{{\varepsilon }_{0}{\chi }_{r}}{2}((-\frac{r}{{\omega }_{0}^{2}}\exp (-\frac{2{r}^{2}}{{\omega }_{0}^{2}})){{\bf{e}}}_{{\boldsymbol{r}}})+s\frac{{\varepsilon }_{0}{\chi }_{i}}{2}((\frac{r}{{\omega }_{0}^{2}}\exp (-\frac{2{r}^{2}}{{\omega }_{0}^{2}}){{\bf{e}}}_{{\boldsymbol{\varphi }}})),$$

where *ε*_0_ is the dielectric constant, *χ* = *χ*_*r*_ + *iχ*_*i*_ is the electric susceptibility and *s* = ± 1 denotes the right- or left-handed circular polarisation. In the simulation, *χ*_*r*_ = 1.156 and *χ*_*r*_ = 0.202 were used according to the experiment, where an azo-polymer poly- Disperse Red 1 methacrylate (pDR1M) thin film target was used^25^. The target is thought to be isotropic and homogeneous. The absorption coefficient is *α* = 1.77 × 10^4^ cm^−1^ at 488 nm. The first and second terms in Equation (4) denote the radial collection force of the beam centre and azimuthal torque, respectively.

**Figure 3:**
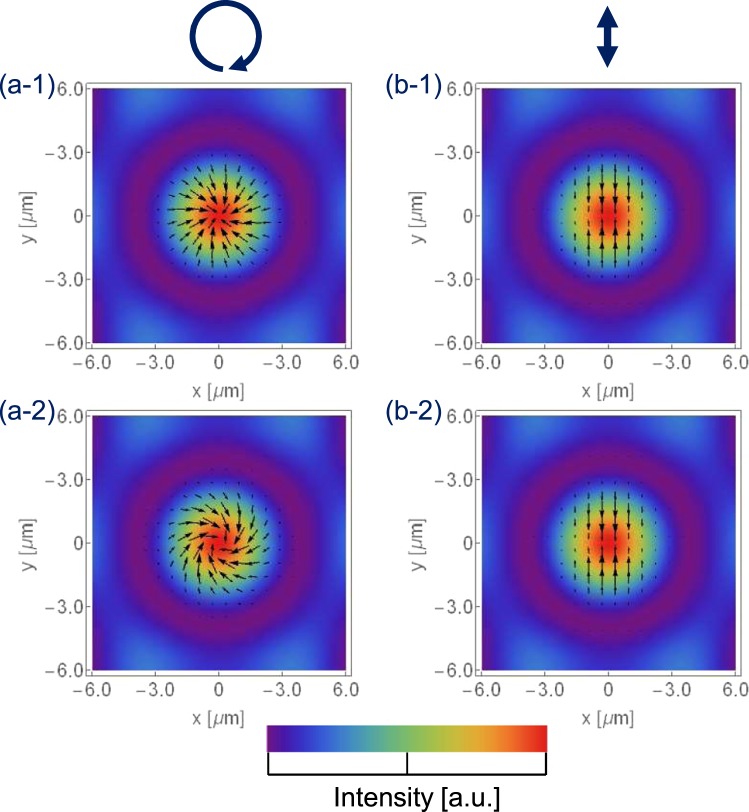
(**a-1**) (**a-2**) Optical radiation force distributions on a spot in a 6-beam interference pattern with right-handed circularly polarised beam; (**b-1**) (**b-2**) optical radiation force distribution with linearly polarised beam. The azimuthal torque is enhanced five times in the case of (**a-2**) and (**b-2**).

Here, we simulate the optical radiation force distribution of a spot in an interference pattern of six beams using the interference pattern shown in Fig. 2 and Equation (4). In the simulation, *s* = 1 and 0 are used according to the experimental condition, where a right-handed circularly polarised beam or linearly polarised beam was used. Figure 3 explains the simulated optical radiation force distributions. The images in the left row show the right-handed circularly polarized beam. The azimuthal torque is considerably less than the radial collection force, so the spiral structure is not evident in Fig. 3(a-1). In the experiment, the spiral structure appears in a time scale of seconds, though a bump structure due to the radial collection force appears within 0.1 second of initiating Gaussian beam irradiation^9^. To visualise the spiral force distribution, the azimuthal torque in Equation (4) is enhanced five times in Fig. 3(a-2). On the contrary, there is no apparent difference in the linearly polarised beam, as shown in Fig. 3(b-1) and (b-2). In all of the following figures, the azimuthal torque is enhanced five times for clarity.

**Figure 4:**
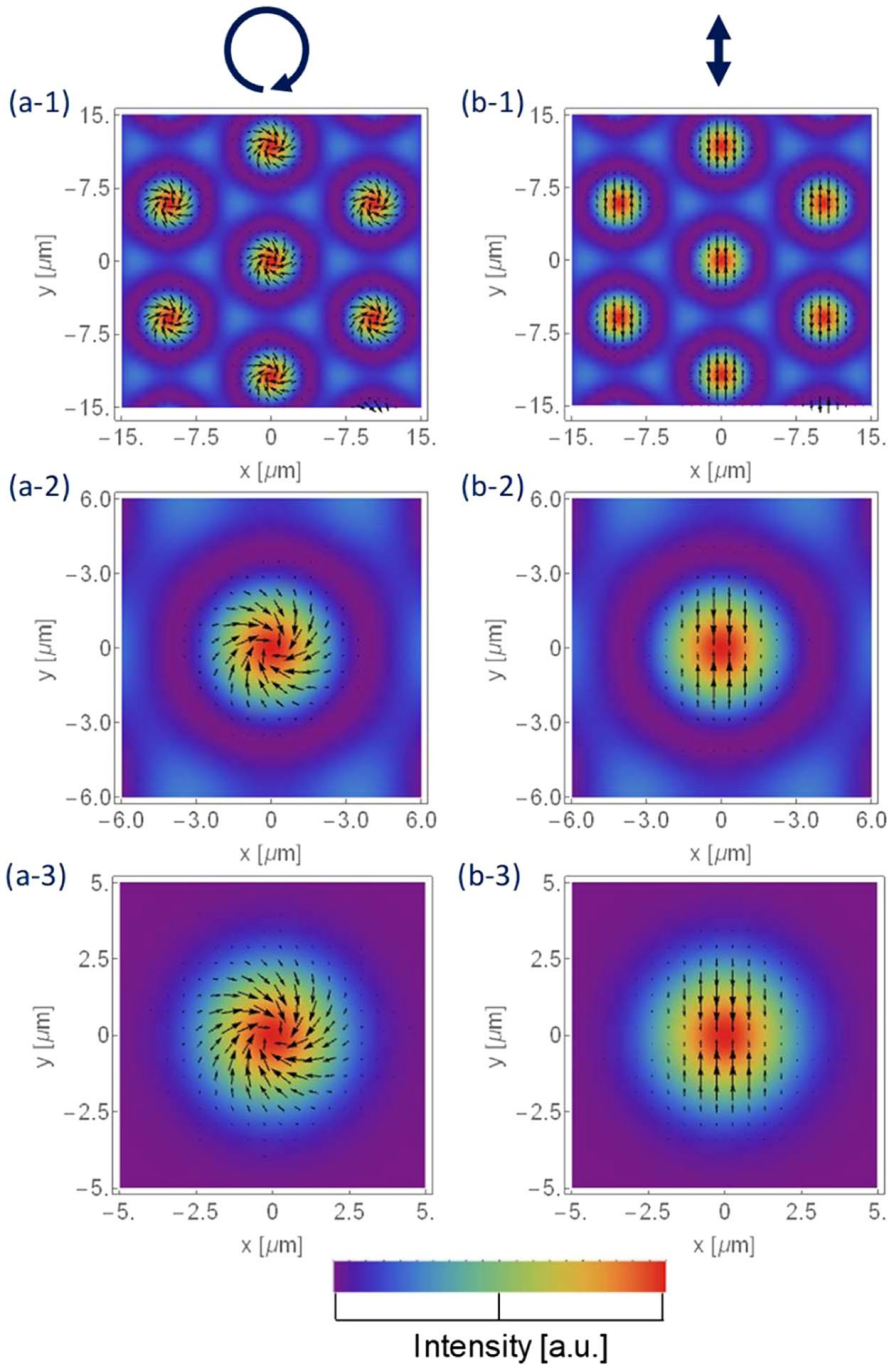
Left row – right-handed circularly polarised beam; right row – linearly polarised beam; (**a-1**) (**a-2**) (**b-1**) (**b-2**) optical radiation force distributions of 6-beam interference pattern, and (**a-3**) (**c-3**) of Gaussian beam.

The optical radiation force distributions of the interference pattern and a Gaussian beam is shown in Fig. 4. Images of the right-handed circularly polarised beam are in the left row and images of the linearly polarised beam are in the right row. The optical radiation force is distributed similarly on all the spots in the interference patterns, as shown in Fig. 4(a-1) and (b-1). In the magnified view of Fig. 4(a-2), the force causes gathering and twisting in the spot area, similar to that in the case of the Gaussian beam shown in Fig. 4(a-3). It should be noted that the optical radiation force at the peak is zero, where there is no power density gradient. Under these conditions, a single spiral structure has been fabricated on the azo-polymer target using the Gaussian beam^9^. On the contrary, the force causes a ridge to form on the spot area in the case of the linearly polarised beam, as shown in Fig. 4(b-2) and (b-3). In summary, the simulation indicates that the force can be distributed to form a chiral or ridge structures in lattice in the case of an interference pattern.

## Results and Discussion

**Figure 5:**
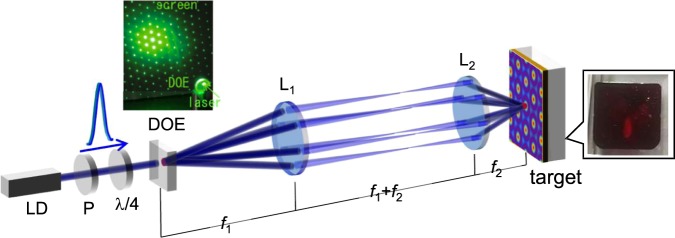
Experimental setup. P: polariser, DOE: diffractive optical element that diffracts six beams equally. *λ*/4: quarter-wave plate. The top left image shows the diffraction pattern of the DOE at *λ* = 532 nm.

The experimental setup is shown in Fig. 5. In this experiment, azo-polymer pDR1M (Sigma-Aldrich) thin film was used^26^. The glass transition temperature was *T*_g_ = 82 °C, the melting temperature was *T*_m_ = 300 °C and the absorption peak wavelength was *λ*_max_ = 467 nm. The film size was 10 × 10 mm and the thickness was measured to be approximately 12 μm. A single-mode CW laser system was operated at 488.0 nm. The beam was split into six first-order diffracted beams and one zeroth-order beam using a diffractive optical element (DOE). The top left image shows the diffraction pattern at *λ* = 532 nm. They interfere on the target surface, and the period of the interference pattern was *Λ* = 11.8 μm.

**Figure 6:**
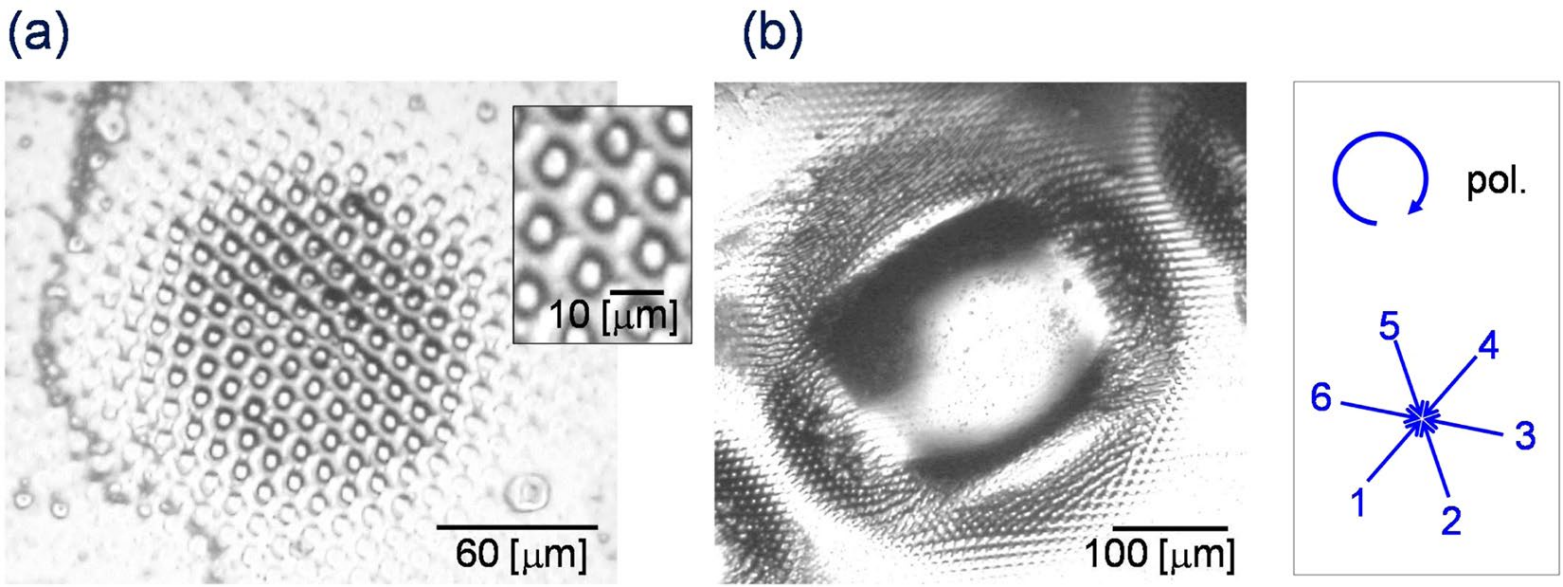
Optical image of the structure fabricated by an interference pattern of six circularly polarised laser beams: (**a**) *P* = 35.8 mW and Δ*t* = 6.0 seconds, (**b**) *P* = 71.6 mW and Δ*t* = 10.0 seconds.

The optical image of the structure fabricated by an interference pattern of the circularly polarised laser beam is presented in Fig. 6. The total laser power and exposure duration was (a) *P* = 35.8 mW and Δ*t* = 6.0 seconds, and (b) *P* = 71.6 mW and Δ*t* = 10.0 seconds. In Fig. 6(a), a regular triangle lattice structure with a lattice constant *Λ* = 11.8 μm was fabricated, which is in accordance with the interference pattern^27^. The corresponding density was 8.29 × 10^5^ cm^−2^. On the contrary, a large molten bump without periodic structure was fabricated at a higher laser power and longer duration, as shown in Fig. 6(b). In this case, the temperature of the corresponding area should be higher than the melting point. In addition, a distorted periodic structure can be seen on the rim, where the power density is relatively lower.

**Figure 7:**
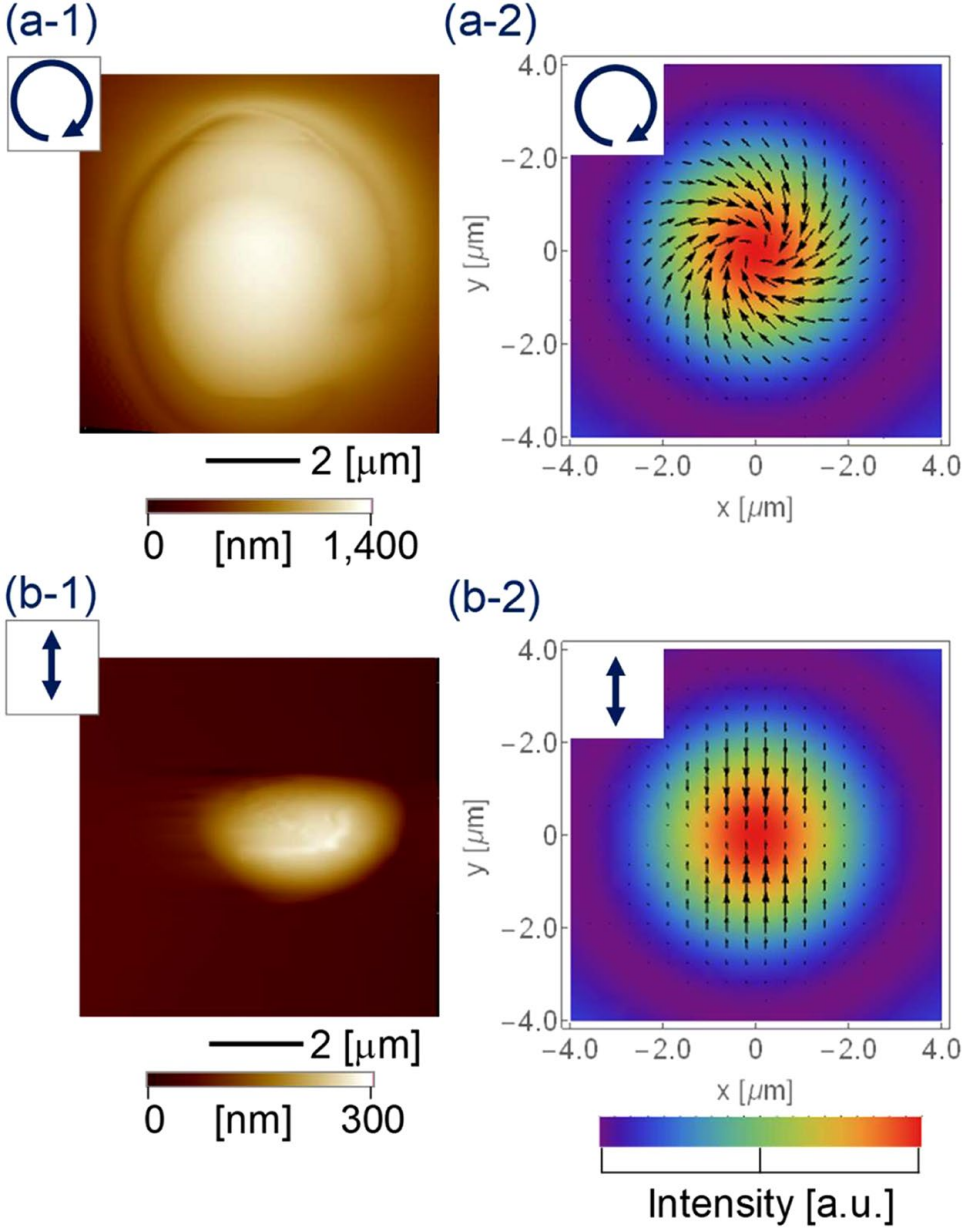
AFM image of the centre spot in a lattice structure fabricated by an interference pattern of (**a-1**) circularly polarised beam or (**b-1**) linearly polarised beam; (**a-2**) and (**b-2**) are the corresponding optical radiation force distributions from Fig. 4.

An atomic-force microscope (AFM) image is presented in Fig. 7 that shows the centre spot in a lattice structure fabricated by an interference pattern of (a-1) the circularly polarised beam or (b-1) the linearly polarised beam. The total laser power was 35.8 mW in both experiments, and the exposure duration was 6.0 and 8.0 seconds, respectively. Assuming the beam radius of the interference pattern on the target was *ω*_0_ = 87.5 μm, the averaged power density at the centre of the spot, where the interference pattern was not considered and the six beams overlapped perfectly, was *I*_center_ = 300 W/cm^2^.

**Figure 8:**
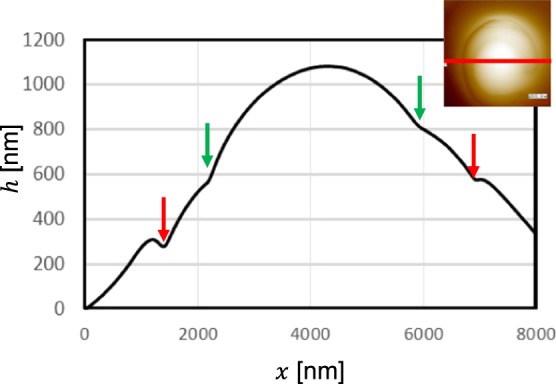
Cross-sectional graph of Fig. 7(a-1) along *x* direction.

For the circularly polarised beam, a right-handed spiral bump structure was fabricated, as shown in Fig. 7(a-1), which was due to the spiral force distribution, as shown in Fig. 7(a-2). The diameter was approx. 5.6 μm and the peak height was 1.1 μm, as indicated by the red arrows in the cross-sectional graph along the *x* direction in Fig. 8. The aspect ratio was 0.2, and the green arrows indicate the border of the spiral step, and the right arrow is higher due to the spirality. In the case of the linearly polarised light, a horizontally long-ridge structure due to the vertically gathering force distribution was fabricated, as shown in Fig. 7(b-1). The lengths of the major and minor axes were 4.3 and 2.8 μm, respectively, and the peak height was 0.3 μm.

**Figure 9:**
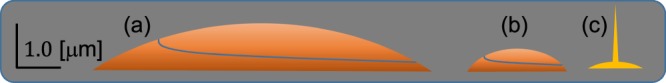
Sketches of the structures: (**a**) spiral in this paper, (**b**) spiral in Omatsu group^9^ and (**c**) gold nanowhisker^11^.

Here, on comparing the morphology with the experiment using a Gaussian beam by Omatsu^9^, where the target was poly-orange Tom 1 azo-polymer and the wavelength was 532 nm, different results can be qualitatively explained as follows. The shapes compared in the sketch are in the same scale in Fig. 9 for an intuitive understanding. The height and diameter in their experiment with the same exposure time were 0.80 and 3.2 μm, respectively, which is approximately 30% of the volume used in this experiment. In addition, the grooves indicated by red and green arrows in Fig. 8 are clearer in their experiment.

These differences could be a result of our process being more photo-thermal due to the weak thermal diffusion in the lateral direction of our area processing, which induced the energy into the neighbouring spots in parallel to interact with each other. In their experiment, the better thermal diffusivity allowed for the use of a power density that was four times higher than that used in our experiment. This could explain the difference in spirality clarity in their experiment. So, the parameters in our experiment should be optimised to enhance the optical radiation force without detriment from the heat. In addition, our spiral structure could be minimised by narrowing the lattice constant of the interference pattern to minimise the spot size. Here, the lattice constant of the interference pattern of the six beams is calculated using

$$A=\frac{2\lambda }{\sqrt{3}\,\sin \,{\theta }_{n}}.$$

For visible light, the minimum lattice constant will be sub-micro in size. On the contrary, the area with a strong optical radiation force and chirality is near the centre of each spot, as shown in Fig. 4. The parameters should be optimised to obtain nano- or sub-micro chiral structures formed by an optical radiation force induced by a circularly polarised beam.

The morphology should also be compared with that of the metal nanostructure fabricated by interference femtosecond laser processing^11,13^. The metal nanostructures are formed via solid-liquid-solid (SLS) process, where thin film is evaporated and launched by the reaction of vapour pressure and/or thermal stress, and a nanodrop is formed by surface tension. At higher fluence, a nanowhisker is formed by pinching off the droplet and quick freezing^11^. The gold nanowhisker has a relatively high aspect ratio of 16.5, as shown in Fig. 9 (c), which is more than 60 times larger than those fabricated by an interference pattern and a Gaussian beam, as illustrated respectively in Fig. 9(a) and (b). The averaged fluence was 136 mJ/cm^2^, which is 1/13200 of the experiment in this paper. These differences come from the different driving forces used to fabricate these concave structures.

## Conclusion

This study demonstrated that the interference pattern of a circularly polarised beam is useful to fabricate a chiral structure in lattice. Due to the nature of the interference, the period is critical and controllable, which leads to the fabrication of a wide-area chiral device and enhances the phenomena that originate from the chirality. From the perspective of the laser processing method, optical radiation force was introduced in interference laser processing for the first time. Under this scheme, interference patterns that use another number of beams are adaptive. In addition, any dielectric material with appropriate absorbance can be used. The field of chiral processing by laser is very new, and most investigations are still undergoing fundamental research or are in the discovery phase. The characteristics, simplicity and adaptability of this method will widen the present field of research and accelerate practical applications, such as chiral metamaterials and chirality sensors.

## Methods

In the simulations of the interference patterns, Equations (1), (2) and (3) are expressed as functions in Wolfram Mathematica. To simulate an interference pattern, Equation (2) is integrated over Δ*t* = *λ*/*c*. In the simulation of optical radiation force distributions using Equation (4), a Gaussian beam profile was assumed for each spot in the interference pattern.

pDR1M (Sigma-Aldrich) in acetone solution was suspended onto plasma-cleaned silica glass substrate (10  ×  10  ×  1.0 mm). Then a drop of toluene was dropped to enhance the spreadability, and dried naturally while being held horizontally in atmosphere.

The experimental setup is shown in Fig. 5. A linearly polarised CW laser beam from a LD (Sapphire SF 488, Coherent) was operated at *λ* = 488 ± 1.0 nm, and the diameter was 0.70 ± 0.05 mm (width at 1/*e*^2^ intensity). The beam passes a polariser to enhance the polarisation. A *λ*/4 plate was inserted to transform the polarisation to right-handed circular.

A DOE (Holo/Or Ltd.) optimised to the wavelengths, which has 71.6% diffraction efficiency, split six first diffraction beams with equal power distribution. The diffraction angle was *θ*_diff_ = 0.634. They were correlated on the surface of the target via a demagnification system consisting of two convex lenses with *F*_1_ = 100 mm and *F*_2_ = 25.0 mm^28,29^. All the experiments were performed in atmospheric conditions at room temperature. The external structures were profiled using an AFM (VN-8000, KEYENCE) and imaged using an optical microscope equipped to it.
